# SmartMoms – a web application to raise awareness and provide information on postpartum depression

**DOI:** 10.1186/s12884-023-05680-9

**Published:** 2023-05-31

**Authors:** Daria Daehn, Claudia Martens, Viola Loew, Luisa Kemmler, Sophie Rudolf, Eileen Kochen, Babette Renneberg, Silke Pawils

**Affiliations:** 1 Department of Clinical Psychology and Psychotherapy, Freie Universitaet Berlin, Habelschwerdter Allee 45, 14195 Berlin, Germany; 2 Department of Medical Psychology, University Hospital Hamburg-Eppendorf, Hamburg, Germany

**Keywords:** SmartMoms, Web app, E-mental-health, Postpartum Depression, Digital psychoeducation

## Abstract

**Background:**

Postpartum depression is a major public health concern, which is associated with negative consequences for both mothers and children. Unfortunately, many affected women neither understand the warning signs of postpartum depression nor do they know where to seek help. The aim of this study was to evaluate the feasibility of SmartMoms, a German mobile web application (web app) designed to inform women about postpartum depression, support them, and provide an easily accessible self-screening instrument.

**Methods:**

After its development, SmartMoms was distributed through healthcare providers and social media. Feasibility was assessed by examining (1) the experience of postpartum women with the web app, (2) user behaviour, and (3) the experience of healthcare providers with the web app and its distribution. A mixed methods approach was used, including online surveys, usage data, and interviews.

**Results:**

Most women used SmartMoms to prevent postpartum depression and rated the web app as good (on average 4.36 out of 5 stars). The majority of women (62.2%) accessing the self-screening instrument showed a risk for postpartum depression (Edinburgh Postnatal Depression scale score **≥** 12). Most providers (n = 12/13) felt supported through SmartMoms in discussing postpartum depression and considered it a useful offer. Suggestions for improvement were provided.

**Conclusions:**

SmartMoms meets the needs and expectations of mothers and healthcare providers interested in postpartum depression but should be further adapted to include more specific support options and additional information for professionals.

**Supplementary Information:**

The online version contains supplementary material available at 10.1186/s12884-023-05680-9.

## Introduction

Pregnancy and childbirth are two significant and challenging events in a woman’s life. The birth of a child is accompanied by enormous changes and new responsibilities, putting the postpartum period at high risk for the onset of depression [1]. Postpartum depression (PPD) is an affective disorder with a global prevalence rate of approximately 17% [2], which develops within the first year after childbirth [3]. A number of risk factors have been associated with the development of PPD, including a history of depression and anxiety, lack of social support, high life stress and marital or partner dissatisfaction [4, 5]. The signs and symptoms of PPD are similar to those of major depression at other life stages – including depressed mood, loss of interest and joy, disturbances in sleep and appetite, decreased concentration, feelings of worthlessness or guilt - but often also including negative thoughts pertaining to the infant or motherhood [6, 7]. If PPD is left untreated, it may have a variety of negative consequences for the mother, child, and the whole family [1]. A systematic review by Slomian and colleagues (2019) highlighted significant direct and indirect negative effects on the mental health of mothers and their child. Negative effects on the mother’s quality of life and social relationships, the child’s emotional and cognitive development, and mother-child bonding are just some of the factors found to be associated with PPD [1].

Therefore, it seems essential to identify and treat PPD as early as possible. However, many women remain undiagnosed and untreated [8]. Data show that the process of seeking help is often perceived as difficult for most women. Barriers such as low trust in health professionals, not knowing about PPD and worrying that their difficulties will not be taken seriously, or the label of being seen as a ‘bad mother’, prevent women from seeking and accepting professional support [9]. Edhborg and colleagues (2005) also point out that shame, guilt and concern about being stigmatised for having a mental illness may explain why many women do not seek help [10]. Thus, interventions aimed at reducing stigma and educating about PPD in the perinatal period should be expanded [8].

In Germany, healthcare providers such as gynecologists, midwives, and general practitioners do not routinely screen women for symptoms of PPD. Furthermore, they rarely educate women about mental health issues related to childbirth due to lack of adequate monetary compensation and time [11]. Alternative ways to educate and screen women are therefore urgently needed.

As the smartphone is the most commonly used device of women around childbirth [12], it offers great potential for PPD education and screening. Therefore, we developed the psychoeducational web app SmartMoms (www.smart-moms.de) with the overarching goal to promote the topic of PPD in women and healthcare providers otherwise unaware or unwilling to discuss and explore psychopathological symptoms around birth. The web app is not only a tool to provide information on PPD, but also a tool to screen for the occurrence of PPD symptoms in its users. After its development, women were informed about SmartMoms through healthcare providers and social media. The feasibility evaluation of the web app was conducted from the perspectives of users and healthcare providers. The aims were to (1) examine the experiences of postpartum women with the web app, (2) examine user behaviour, and (3) examine healthcare providers’ experiences with the web app and its distribution. As such, our research questions were as follows:

1. Experience of postpartum women: How do women in the postpartum period evaluate the usability of the web app and are they satisfied with the web app?
2. User Behaviour: How do users access the web app and which features do they use?
3. Experience of healthcare providers: How do healthcare providers perceive the web app and its distribution?

## Methods

**Fig. 1:**
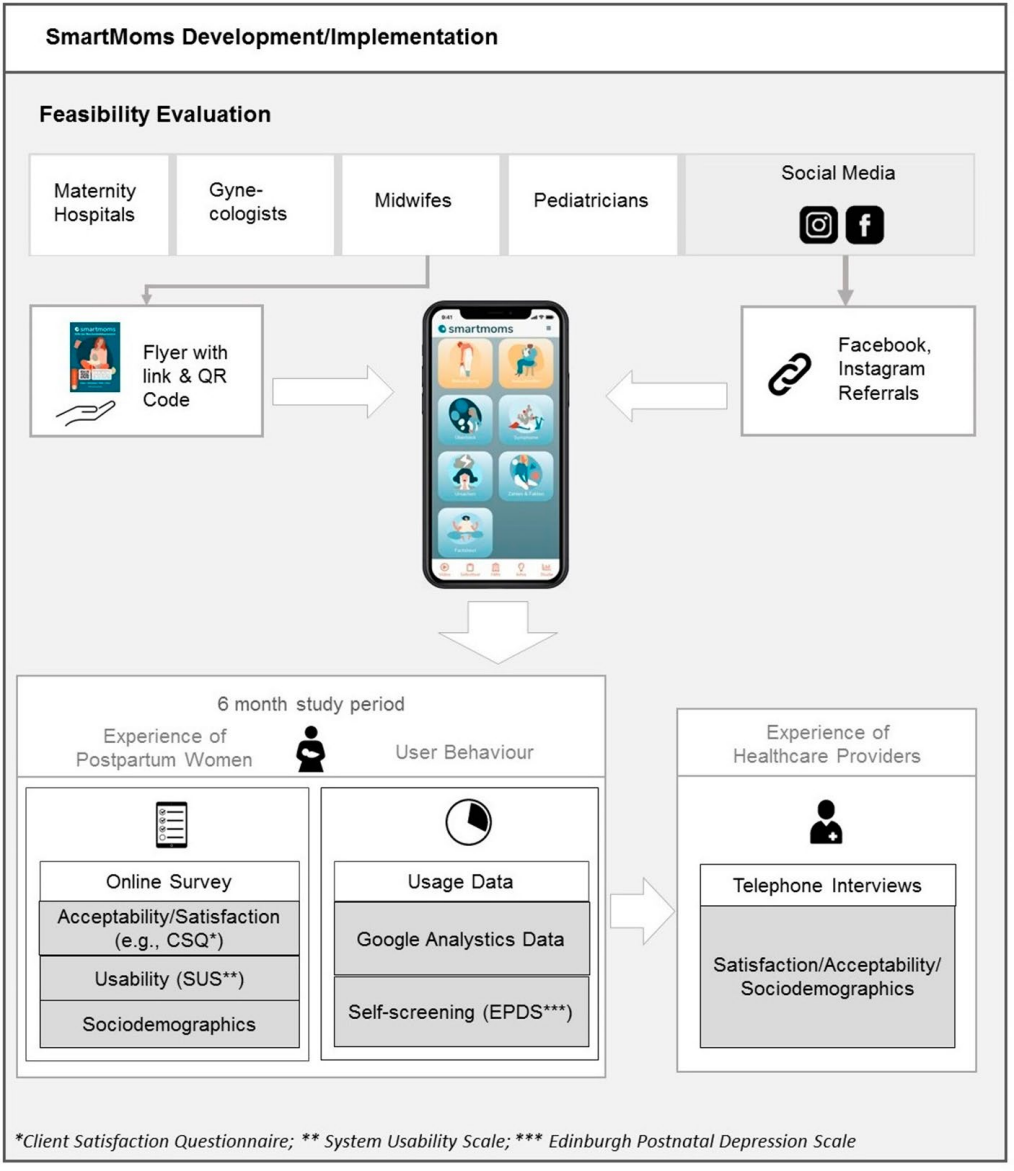
Overview of the Design of the Study

In order to explore the feasibility of SmartMoms, we used a mixed methods approach based on different perspectives (see Fig. 1). For a period of six months (between May and November 2021), the experiences of postpartum women were evaluated by a quantitative online survey integrated into the web app assessing usability and user satisfaction. Additionally, usage data were collected to examine user behaviour such as the engagement with the web app and its features (e.g., self-screening). The experiences of healthcare providers with the web app and its distribution were assessed by telephone interviews after the study period. The study was approved by the local ethics committee of the University Hospital Hamburg Eppendorf (LPEK-0169c) and was funded by Damp Stiftung, Hamburg.

### Intervention

SmartMoms was developed by a team of psychologists and researchers of the University Hospital Hamburg Eppendorf and the Freie Universitaet Berlin in cooperation with an external web app developer. The German web app integrates the following main features:

1. Psychoeducation video with barrier-free content (~ 2 min) informing about PPD symptoms and treatment options
2. Self-screening tool (Edinburgh Postnatal Depression Scale; [13]) allowing women to evaluate their PPD risk
3. General information on PPD (e.g., symptoms, causes)
4. Help page with further support and treatment options

On several subpages, women are encouraged to seek help. To reduce possible feelings of shame associated with PPD, the fact that many women experience emotional difficulties after giving birth, is emphasized [10]. Simple language is used in order to reach a broad range of women. To further simplify usage, audio tracks of the most important content are integrated into the web app. During the development phase, an online survey with 67 female university students was conducted to ensure that the design and colours appealed to women of reproductive age.

**Fig. 2:**
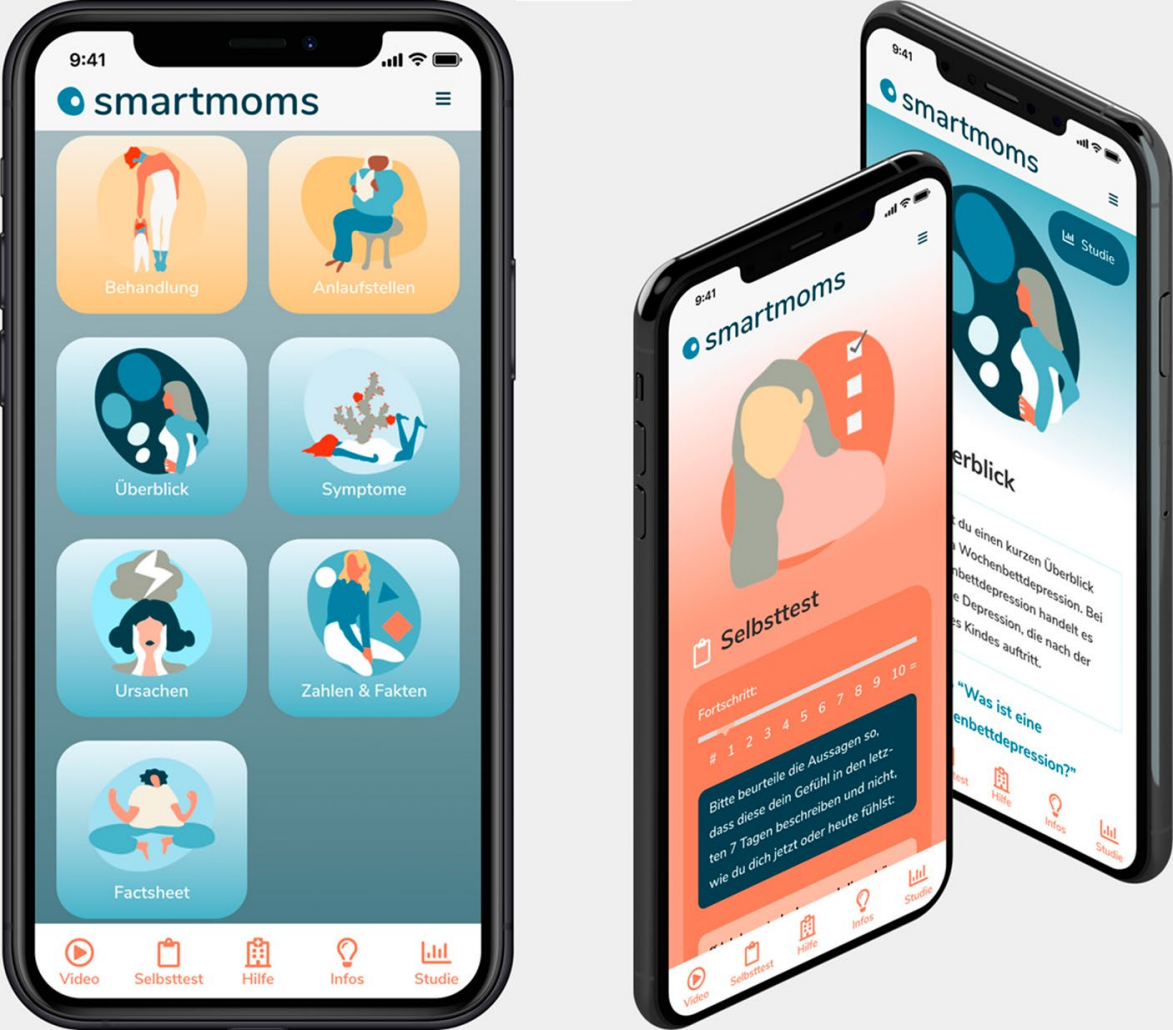
Example of web app interface and content

Upon completion of the self-screening users receive recommendations based on their risk status [13, 14]. We used a cut-off value (EPDS score ≥ 12) as seen in similar studies (e.g., [15, 16]) to avoid false-negative results and to identify most users who meet the diagnostic criteria. Users with scores of 12 or more receive the recommendation to consult a physician, psychotherapist or counselling centre. Additionally, users are encouraged to make use of the support options listed on the *Help Page* of the application. Users deemed at risk of suicide (suicide item of the EPDS) receive the recommendation to immediately contact a professional, call a telephone helpline or, in acute cases, call the emergency service. We also included a high-risk category for users scoring above 20 on the EPDS to stress the importance of contacting a health professional. An overview of the design of the web app is presented in Fig. 2.

### Procedure

To make SmartMoms and the integrated survey accessible to women, we chose two implementation strategies. First, healthcare facilities and providers (e.g., maternity hospitals, midwifes) were recruited in Berlin and Hamburg to inform women about the web app. All participating healthcare providers received SmartMoms flyers (presenting a QR-Code linking to the web app) and were asked to distribute them to their clients for a period of six months. Hospital midwifes, nurses, and doctors were asked to distribute the flyers at the routine medical examination a few days after childbirth. Second, we created two social media accounts (Facebook and Instagram) for user recruitment. On Instagram, we created weekly posts and stories to raise interest in our web app and study among postpartum women. On Facebook, we presented SmartMoms mainly via private groups on postpartum depression or parenting topics and were thus able to recruit further study participants for the evaluation. A total of 117 Facebook groups were contacted for this purpose, of which 28 groups agreed to publish a contribution. An overview of the participating healthcare providers and the digital marketing is presented in Table 1.

Women who had a child in the last 12 months were asked to complete an integrated survey to assess user experiences with the web app. To motivate more women to participate, incentives in form of drugstore vouchers (worth 25–75€) were raffled off among participants. After the study period, health-care providers were contacted, and telephone interviews were conducted to assess their experience with the web app and its distribution among postpartum women.

**Table 1:** Participating healthcare providers/facilities and digital marketing

Healthcare providers/facilities	Hamburg (n = 36)	Berlin (n = 43)
Resident Gynecologists	15	19
Resident Midwifes	9	16
Maternity Hospitals*	7	8
Resident Pediatricians	5	N/A
**Digital Marketing**	**Internet**	
Facebook	117 self-help groups	
Instagram	Weekly content	

**Note**. * At maternity hospitals, physicians, as well as specially trained social workers from the early support intervention program “Babylotse” informed the women.

### Experience of postpartum women

#### Measures

All measures of user experiences were integrated into the web app. A survey consisting of 27 items assessed sociodemographic characteristics, usability of the web app and satisfaction with the web app. Additionally, we asked how women became aware of SmartMoms, why they were using SmartMoms, and whether they were using/are planning to use one of the recommended health services.

#### Usability

The System Usability Scale [17] is a ten-item scale measuring program usability and acceptability. Each item is rated on a five-point scale ranging from one (strongly disagree) to five (strongly agree). An overall SUS Score ranging from zero to 100 can be calculated by: 1.) Calculating the score contribution (ranging from zero to four). For item 1,3,5,7, and 9 the score contribution equals the scale position minus 1. For item 2,4,6,8, and 10 the contribution is 5 minus the scale position; 2) Multiplying the sum of the scores by 2.5. SUS scores can be interpreted as follows: not usable system (score < 65), usable system (65 ≤ score < 85) and excellent system (score ≥ 85) [18].

#### Acceptability and Satisfaction

An adapted version of the Client Satisfaction Questionnaire (CSQ-I; [19]) was used to measure user satisfaction and acceptability. Each of the eight items was rated on a 5-point scale ranging from one (strongly disagree) to five (strongly agree). Higher total scores indicate higher acceptability and satisfaction. Additionally, a self-generated item on overall satisfaction (*“How many stars do you think SmartMoms deserves?”*) was asked (see Results).

#### Analysis

Data were analysed in R [20]. All descriptive statistics are presented as counts, percentages, means and range. Correlational analyses for numerical and binary data (Phi, Spearman’s rho and point biserial correlations) were conducted on sample characteristics and help-seeking intentions using pairwise deletion. Since 14.6% of all datapoints were missing, all following exploratory analyses were conducted on the complete case dataset (n = 125). To explore effects of different sample characteristics (age, marital status, PPD diagnosis, lifetime mental disorder, childbirth experiences) on usability (mean SUS scores) and satisfaction (mean CSQ scores) we conducted multi-factor analyses of variance (type 2). The assumptions of homogeneity of variance and normality of residuals were checked beforehand (Levene’s test and QQ-plot). The variable Marital Status was excluded from all analyses since only one level (partnership) was present in the complete case dataset.

### User behaviour

To analyse user behaviour, we collected:

1. Backend app usage statistics from Google Analytics. Google Analytics can be used as a tool for process evaluation by receiving information on user traffic and subsequently informing website improvement. We analysed user engagement such as number of sessions (total number of visits), users (number of unique visitors) and bounce rate (the percentage of visitors to a particular website who navigate away from the site after viewing only one page). Moreover, we analysed which device (mobile phone, tablet, computer) was used to access the web app. We additionally evaluated whether users accessed the web app by using a direct link (URL, QR-Code), organic search (web search engines such as Google), social media or referral (other websites linking to SmartMoms).
2. Postpartum depression scores were collected via self-screening in the web app (Edinburgh Postnatal Depression Scale; EPDS). The EPDS [13] is a 10-item self-report questionnaire designed to identify mothers at risk for perinatal depression. We assessed all EPDS data anonymously with no possibility to link the information to the user. All users agreed to the use of their data for scientific purposes. As all women interested in the web app were able to have access to the self-screening, some women who were not in the postpartum period also completed the self-screening. We eliminated them from the analyses, as we were only interested in data on women in the postpartum period.

### Experience of healthcare providers

#### Interviews

In order to analyse provider experiences with the content and distribution of the web app, we conducted thirteen structured telephone interviews with midwifes and social workers from October to November 2021. At the time of the interview, healthcare providers had informed postpartum women about SmartMoms for a time of approximately six months. All interested health-care providers received the study information per mail and filled out an informed consent form. With their signature, all participants consented to the audio recording of the interviews. The interviews took approximately 15 min and followed a structured interview guideline.

#### Analysis

We transcribed all interviews after recording. To analyse interview content quantitative and qualitative analyses were combined: All quantitative interview questions were descriptively analysed. All open-end questions or questions, where participants were asked to further describe their responses, were analysed by qualitative content analysis based on Kuckartz [21]. Qualitative analyses were performed on the following questions: (1):* “How did you approach the topic of postpartum depression before participating in the SmartMoms project?”*; (2) *“Has the use of SmartMoms changed your approach?”*; (3) *“What was the response of women to receiving the flyer/link?*”; (4) “*How useful do you consider SmartMoms?*” and (5) *“Which suggestions for improvement do you have*?”. We applied the following six steps recommended by Kuckartz: initial text work, category forming, coding data with main categories, forming subcategories inductively and assigning text passages, category-based analyses, and reporting and documentation. We used MAXQDA 2020 (VERBI Software, 2019) for data analysis. All documents were coded by two independent researchers. Brennan and Prediger’s kappa [22] showed a strong level of agreement (κ = 0.81). All disagreements were solved through discussion.

## Results

### Experience of postpartum women

#### Participants

Descriptive characteristics of participating women (n = 217) are presented in Table 2. The participating women had a mean age of 32.6 years (SD = 4.8, Range = 20–55). The majority of women had one child (M = 1.5; SD = 0.6, Range = 1–4) and lived in a partnership (96.7%). Over half of participating women used SmartMoms for the purpose of PPD prevention. Over 20% of participating women used the web app to assess the personal PPD risk with the included self-screening. 17.8% of participating women had previously been diagnosed with PPD and 22.1% of women had previously used or considered using one of the health services recommended in the web app.

**Table 2:** Descriptive characteristics and frequency distributions of access paths, reasons for using SmartMoms, and help-seeking intentions (n = 217) Note. Based on observed data. a multiple responses possible. Factor levels in parentheses.

Variables	N (%)
**Age in years (n = 214)** *M = 32.5; SD = 4.8; Range = 20–55*	
20–29 (I)	54 (25.2)
≥ 30 (II)	160 (74.8)
**Number of Children (n = 210)** *M = 1.4; SD = 0.6; Range = 1–4*	
1 (I)	136 (64.8)
≥2(II)	74 (35.2)
**Education (Highest degree) (n = 214)**	
None (0)	0 (0)
Lower/Higher Secondary school leaving certificate (I)	61 (28.5)
A level (II)	47 (22.0)
University degree (III)	106 (49.5)
**Marital Status (n = 214)**	
Single (I)	7 (3.3)
Partnership (II)	207 (96.7)
**Lifetime PPD Diagnosis (n = 214)** (*Have you ever been diagnosed with PPD?)*	
No (I)	176 (82.2)
Yes (II)	38 (17.8)
**Lifetime Other Mental Disorder (n = 216)** *(Have you ever been diagnosed with a different mental disorder?)*	
No (I)	194 (89.8)
Yes (II)	22 (10.2)
**Childbirth Experiences (n = 214)** (*How would you describe your birth experience?)*	
Rather negative (I)	138 (64.5)
Rather positive (II)	67 (35.5)
**Help-seeking Intentions (n = 213)** (*Do you already have or plan to use any of the help services mentioned?)*	
No (I)	166 (77.9)
Yes (II)	47 (22.1)
**Access through (n = 216)**	
midwife	12 (5.6)
gynecologist	3 (1.4)
Specially trained social workers	1 (0.5)
Friends	19 (8.8)
Social Media	177 (81.9)
Maternity Clinic	6 (1.9)
**Reasons for using SmartMoms**^**a**^ **(n = 217)**	**% of cases**
Testing vulnerability	44 (20.3)
PPD prevention	130 (59.9)
Getting information anonymously	24 (11.1)
Curiosity	26 (12.0)
Other reasons	34 (15.7)

Note. Based on observed data. ^a^ multiple responses possible. Factor levels in parentheses.

Correlation analyses for numerical and binary data showed a significant and strong positive correlation between: 1) lifetime PPD diagnosis and help-seeking intentions, as well as 2) lifetime diagnosis of a different mental disorder and help-seeking intentions. Specifically, intentions to use one of the recommended health services were more frequently reported by women who had previously been diagnosed with PPD (ϕ = 0.62, *p* < 0.001) and women who have previously been diagnosed with a different mental disorder (ϕ = 0.34, *p* < 0.001). Moreover, a significant and small correlation was found between subjective childbirth experiences and PPD diagnosis. Women who reported negative birth experiences were more likely to report having been diagnosed with PPD (ϕ=-0.18, *p *< 0.05). All other correlational coefficients and associated p values can be found in in Table 3.

**Table 3:** Correlation Matrix for binary and continuous study variables

Variables	1	2	3	4	5	6	7	
1. Age^a^	1							
2. Marital status (I/II) I:Single; II: Partnership	.16*	1						
3. Children^a^	.21**	.02	1					
4. PPD (I/II) I: No; II: Yes	.08	− .02	.10	1				
5. Mental disorder (I/II) I:No; II: Yes	− .03	− .06	.09	.25**	1			
6. Childbirth exp. (I/II) I:Neg.; II: Pos.	.02	− .03	.15*	− .18*	− .01	1		
7. Help-seeking (I/II) I:No; II: Yes	.01	− .03	.04	.62***	.34***	− .18*	1	

Note. Correlations for cross tables for 2 × 2 tables are phi coefficients; correlations between continuous variables are spearman’s rho coefficients, correlations between continuous and binary variables are point biserial. ^a^ continuous variables, all others are binary *p < .05. **p < .01.*** p < .001. Correlation coefficients were based on pairwise deletion.

#### Usability

129 users completed all items on the System Usability Scale (SUS). The individual item scores are presented in Table 4. A total SUS score of 75.20 showed a good usability of SmartMoms [23].

**Table 4:** Usability Evaluation

Variables	M(SD)^a^	Range
System usability scale (SUS) items (n = 129)		
(1) I think I would like to use SmartMoms frequently	2.39 (0.85)	0–4
(2) I found SmartMoms unnecessarily complex*	2.91 (0.95)	0–4
(3) I thought SmartMoms was easy to use	3.12 (0.78)	0–4
(4) I think that I would need the support of a technical person to be able to use this system*	3.36 (0.85)	0–4
(5) I found the various functions in this system were well integrated	2.97 (0.78)	0–4
(6) I thought there was too much inconsistency in this system*	2.98 (0.77)	1–4
(7) I would imagine that most people would learn to use this system very quickly	3.13 (0.77)	0–4
(8) I found the system very cumbersome to use*	3.12 (0.76)	0–4
(9) I felt very confident using the system	2.88 (0.89)	0–4
(10) I needed to learn a lot of things before I could get going with this system*	3.22 (0.84)	1–4
**Total SUS score (n = 129)**	**75.20**	N/A

**Note.** Based on observed data. * Recoded values ^a^ SUS converted score (0–4)

An overview of the effects of the various participant characteristics on mean ratings of web app usability (Mean SUS score) with all associated parameter estimates can be found in Additional file 1 (Table S1). The effect of PPD diagnosis yielded an F ratio of F (1, 117) = 7.63, *p* =.007, indicating that women who had been previously diagnosed with PPD rated the usability of SmartMoms significantly higher (M = 3.54; SD = 0.43) than women without PPD diagnosis (M = 3.01; SD = 0.53). All other effects were non-significant.

#### Acceptability and Satisfaction

133 users completed all items of the adapted CSQ on web app satisfaction and acceptability (see Table 5). Most participants agreed or strongly agreed with the statement *“In an overall, general sense, I am satisfied with SmartMoms”* (79.7%) and with the statement *“I would recommend SmartMoms to a friend if she was in need of similar help”* (79.0%). To measure overall satisfaction, we additionally asked participants to rate the web app on the self-generated question *“All in all, how many stars (1–5) do you think SmartMoms deserves?”. Overall, SmartMoms obtained high ratings* (M=4.36; SD = 0.77).

**Table 5:** User Satisfaction and Acceptability (n = 133)

Variables	M(SD)	Range
**Client satisfaction questionnaire (CSQ) items (n = 133)**		
(1) SmartMoms was of high quality	3.86 (0.77)	2–5
(2) I received the kind of information I wanted to	3.99 (0.77)	2–5
(3) SmartMoms has met my needs	3.88(0.77)	2–5
(4) I would recommend SmartMoms to a friend if she was in need of similar help	4.15(0.80)	2–5
(5) I am satisfied with the amount of recommendations I received	3.99(0.77)	2–5
(6) SmartMoms helped me deal with my problems effectively	3.62(0.79)	2–5
(7) In an overall, general sense, I am satisfied with SmartMoms	4.05(0.67)	3–5
(8) I would come back to SmartMoms if I would need help again	4.07 (0.75)	2–5
**Overall satisfaction (n = 200)**		
All in all, how many stars (1–5) do you think SmartMoms deserves?	4.36 (0.77)	2–5

Note. Based on observed data. * recoded values

An overview of the effects of the various participant characteristics on satisfaction with the web app (Mean CSQ score) with all associated parameter estimates can be found in Additional file 2 (Table S2). The effect of PPD diagnosis yielded an F ratio of F (1, 117) = 9.03, *p* = .003, indicating that women who had already been previously diagnosed with PPD were significantly more satisfied with the web app (M = 4.53; SD = 0.58) than women without PPD diagnosis (M = 3.89; SD = 0.59). All other effects were non-significant.

### User behaviour

During the study period of six months, n = 1874 unique users had 2752 sessions (see Table 6). Overall, the bounce rate of 27.1% was low [24]. Of the content pages the page *Self-Screening* was accessed the most frequently. Although less frequently accessed, the *Help Page*  showed the highest retention time. Specifically, users viewed the subpage *Support Options* longer than any other page. Most users (75.8%) accessed SmartMoms with a mobile phone. 23.0% of users accessed the web app with a computer and 1.2% with a tablet.

**Table 6:** User engagement with the web app (n = 1874)

Activities	No (%)
Number of sessions^a^	2752
Number of unique users^b^	1874
Duration of sessions in minutes^c^	02:32
Bounce rate^d^	(27.1)
First-time visitors^e^	1837 (82.1)
Returning visitors^f^	400 (17.9)
Most visited pages in page views	12.657
Landing Page	3702 (29.3)
Survey Page	1931 (15.3)
Self-screening Page	1906 (15.1)
Information Page	688 (5.4)
Help Page	655 (5.2)
Help-page – Support Options	598 (4.7)
Video Page	463 (3.7)
Mean Time spent on pages in minutes	00:42
Help-page – Support Options	01:07
“About Us” Page	00:59
Survey Page	00:58
Landing Page	00:49
Self-screening Page	00:48
Video Page	00:37

**Note**. ^a^ Period of time users are actively engaged with the web app

^b^ Users who have had at least one session within the selected date

range, includes both new and returning users.

^c^ The average length of a session.

^d^ Visits in which the user left the site from the entrance

page without interacting with the page

^e^ An estimate of the percentage of first-time visits.

^f^ Percentage of visitors returning to the website.

**Fig. 3:**
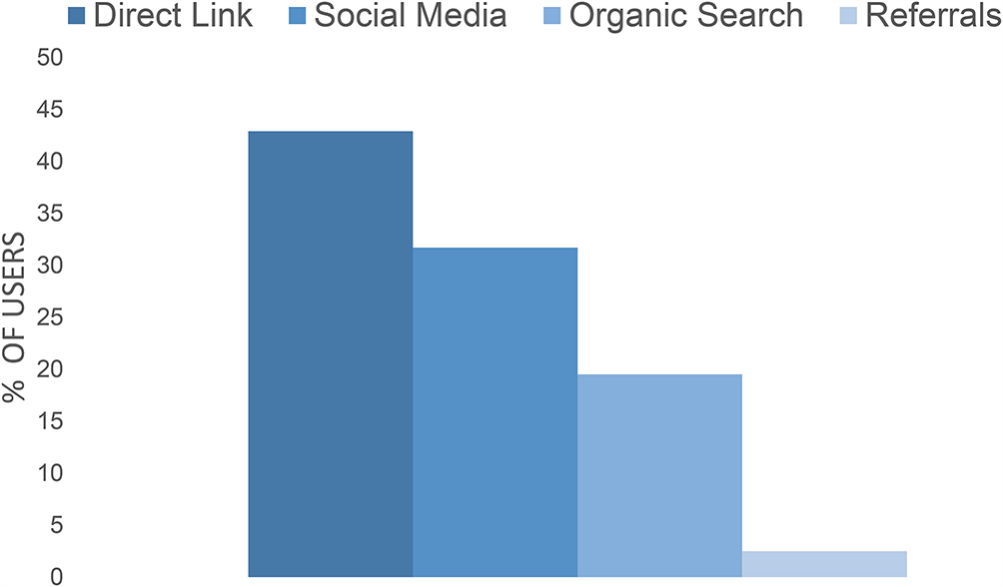
How users accessed SmartMoms

In the six month study period, most users accessed the web app by using a direct link (including QR-Code) (n = 815; 42.9%), followed by Facebook/Instagram referrals (n = 603; 31.7%), organic search (web search engines such as Google) (n = 435; 19.5%) and other referrals (n = 49, 2.5%). A visualization of this can be seen in Fig. 3.

241 women completed all items of the self-screening as measured by the EPDS and provided information on their child’s age. Women with children over 12 months of age have been excluded from the analysis (n = 19) resulting in data of 222 women. The mean EPDS score was 13.4 points where a majority of women (62.2%) showed a risk for PPD (EPDS ≥ 12). All available data on EPDS scores are presented in Table 7.

**Table 7:** Data of women completing the EPDS during the study period of 6 months (n = 222)

Variables	Mean (SD)	Range
EPDS score	13.4 (5.8)	0–28
Maternal Age (n = 219)	32.7 (4.0)	22–42
Child Age (Months)	3.56 (3.7)	0–12
	**N**	%*
EPDS frequency distributions	**222**	**100**
0–11 (PPD rather unlikely)	84	37.8
12–20 (depression likely)	116	52.3
21–30 (depression very likely)	22	10

Note. EPDS (Edinburgh Postnatal Depression Scale). * Sums exceeding 100% by rounding the decimals.

### Experience of healthcare providers

#### Descriptive Statistics

Thirteen professionals (eight specially trained social workers and five midwifes) participated in the interviews. Among the providers who indicated that they felt responsible for the care of women with PPD, two indicated more explicitly that they felt responsible for identifying PPD but not for treating it. Most participants distributed SmartMoms flyers to women who were in the postpartum period. Overall, participating providers viewed SmartMoms as a supportive tool for the discussion of PPD (4.1 / 5 points) and rated the web app as good (4.1 / 5 points). All descriptive statistics are presented in Table 8.

**Table 8:** Descriptive Characteristics of interviewed healthcare providers (n = 13)

Variables	N	%
**Age**		
Mean (SD)	41.6 (9.4)	
Range	27–50	
**Gender**		
Female	13	100
**Profession**		
Midwifes	5	38.5
Specially trained social workers	8	61.5
**Years of professional experience in the specified profession**		
Mean (SD)	6.5 (6.9)	
Range	0.25–20	
**Felt responsibility for the care of women with PPD**		
Yes, IT feel responsible	11	84.6
No, I do not feel responsible	2	15.4
**Timing of flyer distribution**		
Prepartal	3	23.1
Postpartal	8	61.5
Not at all	1	7.7
Not specified	1	7.7
Support felt through SmartMoms (1–5)*		
Mean (SD)	4.1 (1.1)	
Range	1–5	
Overall rating (1–5)*		
Mean (SD)	4.1 (1.1)	
Range	1–5	

**Note.** *Higher ratings indicating higher felt support / higher overall satisfaction

#### Qualitative Content Analyses

Based on the interview questions the following five main categories were deductively formed:

1. Approach towards PPD before using SmartMoms.
2. Changes in approach due to SmartMoms.
3. Women’s responses to SmartMoms.
4. Perceived usefulness of SmartMoms.
5. Suggestions for improvement.

In the following, we present the results for each main category.

#### Approach towards PPD before using SmartMoms

Most healthcare providers (n=8/13) stated that they had informed women about PPD in a personal conversation in case of suspicion. Most of them (n=5/8) additionally used a screening questionnaire. Two out of 13 providers indicated that they held a personal conversation and used a screening questionnaire with every woman, regardless of whether they appeared susceptible to PPD. Three out of 13 providers indicated that they generally did not provide information about PPD.

"*Honestly, not at all. So, when you had a woman, you checked whether you had to address something *… *However, I would also say ... that I have never had such a case."*

#### Changes in Approach due to SmartMoms

As most interviewed healthcare providers were already informing women about PPD, most of them stated that they did not change their approach due to SmartMoms (n=8/13). Five out of 13 indicated that they changed their approach by informing women the first time through flyers or personal conversations or by starting to have personal conversations with all women, regardless of whether they appeared susceptible. Four providers stated that SmartMoms made it easier to address the issue. SmartMoms was used as a conversation starter or the integrated self-screening was used together with the women instead of the paper pencil version.

"*… maybe I just let them go before and did nothing. And now I give them the flyer so that they can just check it out for themselves.*"

#### Women’s responses to SmartMoms

Nine providers stated that they were getting positive feedback from women on receiving the SmartMoms flyers. Specifically, providers described women as being open to the offer and as being grateful or relieved.

"*… They are very open and usually accept it very gratefully*."

Moreover, healthcare providers indicated that women valued that SmartMoms was easily accessible/non-stigmatizing and that they felt empowered through SmartMoms.

" … It’s actually very positive. Because it is somehow … not something that stigmatizes ... "

"*… they can be active themselves* ... "

Three healthcare providers received no feedback from women and one received feedback of irritation.

"*They are irritated. They are already scared anyway …*"

#### Perceived Usefulness of SmartMoms

Twelve out of thirteen considered SmartMoms a useful offer, one stated that she could not estimate whether it was useful. Healthcare providers most often valued that SmartMoms was a low-threshold possibility for PPD education and that SmartMoms was a digital offer. In addition, the clarity of the web app content, and the possibility of using SmartMoms as a complimentary tool were described as useful.

"*I believe that … admitting that it could be postpartum depression is always a challenge. And I believe that this can be a very low-threshold path, which is why I think it is very good.*"

"*I think it is definitely good. Above all, it is also a digital offer and many women are still quite young and very active on the internet … and I think that it can be a good access*."

#### Suggestions for improvement

Six healthcare providers made suggestions for improvement. Four of them mentioned that the support options SmartMoms provides, should be more specific. Moreover, they wished for more additional information for professionals, different languages and a better structure of the web app.

“… *So, when I look at the counselling centres, it’s very broad … I can imagine that it would be easier if [Smartmoms] already suggested places, depending on where you live.*”

## Discussion

New mothers have a great need for information around childbirth [25, 26], which should include information about mental health in the postpartum period [27]. The purpose of this study was to investigate the feasibility of SmartMoms, a psychoeducational web application that provides information on PPD and screening. For this purpose, we examined (1) the experiences of postpartum women with SmartMoms, (2) user behaviour, and (3) healthcare providers’ experiences with SmartMoms and its distribution.

The experiences of women with SmartMoms, as assessed by an online survey, were mainly positive. This was evidenced by high ratings on satisfaction with SmartMoms (mean overall satisfaction score of 4.36 out of 5**)** and a total system usability score of 75.20 out of 100. Interestingly, the only factor affecting usability and satisfaction ratings was PPD diagnosis. Women who had previously been diagnosed with PPD were significantly more satisfied with SmartMoms and rated its usability higher than women without a PPD diagnosis. It is quite possible that these women considered the content of SmartMoms more relevant than women who had no former experience with PPD. While the term usability refers to the interaction between user and system functions, usefulness refers to the interaction between user and content [28]. Previous research has shown that ratings of usefulness and usability are related, which might explain the higher usability ratings of women with previous PPD diagnosis [28]. Research by Slomian et al. also showed that most postpartum women use the internet to learn about themselves or their baby, irrespective of socioeconomic status or age [29]. Thus, it is important that the PPD information found on the internet not only corresponds to scientific findings, but also is accepted by a wide range of women. Satisfaction and usability of the web app should therefore ideally not be affected by factors such as women’s age. Given that the age of users had no significant effect on the usability or satisfaction ratings of survey participants, SmartMoms proves to be suited for women regardless of age. Likewise, the education level of survey participants did not affect the ratings. However, as all participating women had at least a secondary school education level, we do not know whether the web app is also suitable for women with a lower educational level.

Almost 60% of users participating in the survey indicated that they used SmartMoms to prevent PPD. Approximately 20% of users indicated to use the web app to screen for symptoms of PPD. Interestingly, almost 75% of users who used the self-screening tool (Edinburgh Postnatal Depression Scale, [13]) presented a risk or a high risk for PPD. This shows that the self-screening tool was mostly used by women with postpartum depressive symptoms. Usage data showed that apart from the landing and the survey pages, users most frequently accessed the self-screening and information pages of the web app. In addition, most users accessed SmartMoms on their smartphone and almost 43% of users accessed the website by opening the URL/QR-code directly. This suggests that many users received a recommendation for SmartMoms from their healthcare provider. Another 32% of users accessed the web app through Facebook/Instagram referrals, indicating that the users can be reached via both healthcare providers and social media. In the survey, however, nearly 82% of participating women reported that they found the link to the web app on Facebook or Instagram. This suggests that while many women who used the web app received the link/code from their healthcare provider, these women were not as likely as women recruited from social media to participate in the survey. This discrepancy underscores the importance of social media engagement for the collection of survey data when evaluating information technologies. However, it should not be overlooked, that healthcare providers appear to play a major role in the distribution of educational and or screenings tools for women in the postpartum period.

As recommended by Pawils et al. (2022), the basis for adequate management of PPD should consist of routine screening of postpartum women by primary care providers [11]. Women in Germany are generally well integrated into the healthcare system during and after pregnancy and are in contact with healthcare providers such as midwives, gynecologists, pediatricians and general practitioners [30, 31]. Thus, these healthcare providers play an important role in the diagnosis of PPD and in the referral to further mental health services. Unfortunately, due to the limited consultation time of healthcare providers as well as the perceived lack of adequate monetary compensation, information on PPD and screening are often not provided [11]. Therefore, it was important to investigate whether SmartMoms supports healthcare providers in screening women or educating them about PPD. Most of the healthcare providers interviewed viewed the web app as useful and emphasized the fact, that it is a low-threshold tool and provides information digitally. Some healthcare providers that had previously not informed about PPD stated that they had started talking to women about the issue. Moreover, some providers reported that they beganinforming more women than they had in the past. Others reported that SmartMoms facilitated educational conversations and served as a conversation starter. Therefore, SmartMoms appears to help health care providers talk to postpartum women about PPD. This is underscored by an average rating of 4.1 out of 5 for the web app. Besides, most healthcare providers received positive feedback from the women receiving the web app flyers, which strengthens our quantitative survey results. They reported that women were grateful and valued that the web app was empowering, easilyaccessible and not stigmatizing. As some providers already recommended SmartMoms during pregnancy, a follow-up survey should examine the value of using SmartMoms for PPD education before birth. In practice, gynecologists could use the web app with all postpartum women as part of their follow-up appointments and have their patients take the self-screening while still in the waiting room. They could then use the web app and the self-screening as a conversation starter to refer affected women to more qualified care, if necessary. To facilitate the procedure in practice, the web app is currently being expanded on the basis of the suggestions of the interviewed healthcare providers to include a sub page for professionals (including tips on how to start a conversation, step-by-step guide) and more specific support options.

As an interesting additional finding, we found a correlation between subjective childbirth experience and PPD diagnosis. This finding may point in the direction of previous research suggesting that a negative subjective birth experience may contribute to postpartum depression (for a review see Bell & Andersson, 2016 [32]). However, it still remains unclear what exactly characterizes a negative subjective birth experiences: in particular, it is still unknown which moments during birth are experienced as most stressful and which role interpersonal strain and support play during these moments. Future studies should investigate interpersonal strain and support in the most stressful moments during birth, especially with regard to their impact on postpartum mental health.

### Strengths and limitations

To the best of our knowledge, this was the first study evaluating the feasibility of a psychoeducational web app offering information and screening on PPD in Germany. A strength of the study is that the web application was tested in a real-world environment, allowing the usage data to provide insight into real-world usage. Another strength is that we used different distribution strategies (various healthcare providers informing about the web app and distributing flyers, and social media). Thus, by analysing user behaviour the study provided profound insights on how to reach women in the postpartum period. Moreover, by assessing the experiences of both women and providers, as well as analysing usage data, we obtained a comprehensive picture of the feasibility of the web app. The qualitative data obtained from healthcare providers (midwives and social workers) allowed us to gain insights into how web applications on PPD can support providers and what improvements should be considered. It should be noted, however, that we were unable to recruit gynecologists for the interviews and that our sample size was small. Additionally, we must assume that only providers with an awareness of the topic agreed to use SmartMoms to educate women about PPD. Therefore, our findings are not representative and cannot be generalized to all healthcare providers working with postpartum women. As this is a feasibility study focusing on the experiences of users and healthcare providers, a limitation is that we cannot draw any conclusions on the effectiveness of the web app in terms of mental health literacy, self-efficacy, or help-seeking behaviour. These constructs are currently being investigated in a follow-up study. Moreover, it has to be stressed that our survey results cannot be generalized, as respondents made up only a small proportion of web app users. Lastly, as a psychoeducational web app, SmartMoms can only act as a first step in PPD care. Therefore, the findings from this project will be used to develop and evaluate an application especially for fathers and a mobile cognitive-behavioural intervention for women with postpartum depressive symptoms in Germany in the follow-up project Smart-e-moms, that will be funded by the Innovation Fund of the Federal Joint Committee (G-BA starting in 2023).

## Conclusion

The German web app SmartMoms, which provides information and screening for PPD, proved to be a well-accepted and useful digital tool for both women and healthcare providers interested in postpartum depression. The integrated self-screening could be used as a complementary solution for healthcare professionals to screen women for symptoms of PPD more efficiently.

## Electronic supplementary material

Below is the link to the electronic supplementary material.

Additional file 1: Table S1. Overview of the effects of the various participant characteristics on mean ratings of web app usability (Mean SUS score) with all associated parameter estimates

Additional file 2: Table S2. Overview of the effects of the various participant characteristics on satisfaction with the web app (Mean CSQ score) with all associated parameter estimates

## Data Availability

The datasets used and/or analysed during the current study are available from the corresponding author on reasonable request.

## Abbreviations

PPD: Postpartum depression
EPDS: Edinburgh Postnatal Depression Scale
App: Application
