# Supplementary material for: SmartMoms – a web application to raise awareness and provide information on postpartum depression
Source: BMC Pregnancy Childbirth. 2023 May 31;23:402. doi: 10.1186/s12884-023-05680-9 (PMC10230750; doi:10.1186/s12884-023-05680-9)
Supplement: Supplementary file 1 — Additional file 1: Table S1. Overview of the effects of the various participant characteristics on mean ratings of web app usability (Mean SUS score) with all associated parameter estimates [file 12884_2023_5680_MOESM1_ESM.docx]

*Table S1. Effects of different participants' characteristics on mean ratings of Web app usability (Mean SUS score)*

|  | Mean (sd) | Sum of squares | df | F | p |
| --- | --- | --- | --- | --- | --- |
| **Age**  20-29 (I)  >=30 (II) | 3.11 (.49)  3.02 (.55) | 0.223 | 1 | 0.792 | *.375* |
| **Children**  1 (I)  2-4 (II) | 3.03 (.51)  3.08 (.57) | 0.008 | 1 | 0.028 | *.867* |
| **Education**  Sec. school (I)  A level (II)  University (III) | 3.04 (.54)  3.04 (.61)  3.05 8.51) | 0.040 | 2 | 0.071 | *.932* |
| **PPD diagnosis**  No (I)  Yes (II) | 3.01 (.53)  3.54 (.43) | 2.145 | 1 | 7.629 | *.007*  **** |
| **Psych. disorder**  No (I)  Yes (II) | 3.03 (.54)  3.29 (.55) | 0.007 | 1 | 0.025 | *.875* |
| **Childbirth exp.**  Rather Neg. (I)  Rather Pos. (II) | 3.02 (.54)  3.06 (.54) | 0.071 | 1 | 0.252 | *.617* |
