# Supplementary material for: SmartMoms – a web application to raise awareness and provide information on postpartum depression
Source: BMC Pregnancy Childbirth. 2023 May 31;23:402. doi: 10.1186/s12884-023-05680-9 (PMC10230750; doi:10.1186/s12884-023-05680-9)
Supplement: Supplementary file 2 — Additional file 2: Table S2. Overview of the effects of the various participant characteristics on satisfaction with the web app (Mean CSQ score) with all associated parameter estimates [file 12884_2023_5680_MOESM2_ESM.docx]

*Table S2. Effects of different participants' characteristics on satisfaction with the Web app (Mean CSQ score)*

|  | Mean (sd) | Sum of squares | df | F | p |
| --- | --- | --- | --- | --- | --- |
| **Age**  20-29 (I)  >=30 (II) | 4.08 (.52)  3.88 (.63) | 0.643 | 1 | 1.841 | *.178* |
| **Children**  1 (I)  2-4 (II) | 3.97 (.57)  3.88 (.68) | 0.400 | 1 | 1.144 | *.287* |
| **Education**  Sec. school (I)  A level (II)  University (III) | 3.97 (.59)  4.00 (.64)  3.90 (.61) | 0.130 | 2 | 0.186 | *.830* |
| **PPD diagnosis**  No (I)  Yes (II) | 3.89 (.59)  4.53 (.58) | 3.154 | 1 | 9.029 | *.003*  **** |
| **Psych. disorder**  No (I)  Yes (II) | 3.92 (.60)  4.21 (.76) | 0.024 | 1 | 0.069 | *.793* |
| **Childbirth exp.**  Rather Neg. (I)  Rather Pos. (II) | 3.94 (.61)  3.94 (.61) | 0.053 | 1 | 0.152 | *.698* |
